# Mn-Induced Thermal Stability of L1_0_ Phase in Fept Magnetic Nanoscale Ribbons

**DOI:** 10.3390/nano10071278

**Published:** 2020-06-30

**Authors:** Alina Daniela Crisan, Aurel Leca, Dan Pantelica, Ioan Dan, Ovidiu Crisan

**Affiliations:** 1 National Institute for Materials Physics, P.O. Box MG-7, 077125 Magurele, Romania; alina.crisan@infim.ro (A.D.C.); aurel.leca@infim.ro (A.L.); 2 National Institute for Physics and Nuclear Engineering, P.O. Box MG-6, 077125 Magurele, Romania; dpantelica@yahoo.fr; 3 R&D Consulting and Services S.R.L., 023761 Bucharest, Romania; ioan_dan@rd-consultanta.ro

**Keywords:** nanocomposite magnets, L1_0_ FeMnPt, structural stability, temperature-dependent X-ray diffraction

## Abstract

Magnetic nanoscale materials exhibiting the L1_0_ tetragonal phase such as FePt or ternary alloys derived from FePt show most promising magnetic properties as a novel class of rare earth free permanent magnets with high operating temperature. A granular alloy derived from binary FePt with low Pt content and the addition of Mn with the nominal composition Fe_57_Mn_8_Pt_35_ has been synthesized in the shape of melt-spun ribbons and subsequently annealed at 600 °C and 700 °C for promoting the formation of single phase, L1_0_ tetragonal, hard magnetic phase. Proton-induced X-ray emission spectroscopy PIXE has been utilized for checking the compositional effect of Mn addition. Structural properties were analyzed using X-ray diffraction and diffractograms were analyzed using full profile Rietveld-type analysis with MAUD (Materials Analysis Using Diffraction) software. By using temperature-dependent synchrotron X-ray diffraction, the disorder–order phase transformation and the stability of the hard magnetic L1_0_ phase were monitored over a large temperature range (50–800 °C). A large interval of structural stability of the L1_0_ phase was observed and this stability was interpreted in terms of higher ordering of the L1_0_ phase promoted by the Mn addition. It was moreover found that both crystal growth and unit cell expansion are inhibited, up to the highest temperature investigated (800 °C), proving thus that the Mn addition stabilizes the formed L1_0_ structure further. Magnetic hysteresis loops confirmed structural data, revealing a strong coercive field for a sample wherein single phase, hard, magnetic tetragonal L1_0_ exists. These findings open good perspectives for use as nanocomposite, rare earth free magnets, working in extreme operation conditions.

## 1. Introduction

There is nowadays a sustained research interest for developing new magnetic materials without rare earth elements (RE), while taking into account the critical raw materials shortage of these materials and their contaminating effects on the environment. The European Union, through its EU Raw Materials Initiative [1], has warned about the fact that several raw materials, including heavy and light RE, are critical for the EU because risks of supply shortage, and their impacts on the economy are higher than those of most of the other raw materials. Especially, the end-of-life recycling input rate for rare earths is listed to be only at the level of 3% [2]. As a response to these challenges, the research community is searching for alternatives for RE-free magnets. Among the investigated systems, the alloys exhibiting L1_0_ tetragonal phase, such as FePt or ternary alloys derived from FePt, show most promising magnetic properties. FePt alloys are also of great interest because of their high operating temperatures—higher than that of Nd-Fe-B magnets—and their high resistance to corrosion [3]. A technological barrier that impedes the adoption of such systems is related to the fact that the tetragonal, hard magnetic L1_0_, the phase of interest, is only formed after post-synthesis annealing of the alloys [4]. Several attempts to circumvent this barrier included slight stoichiometry changes in the initial binary alloys or the addition of several elements such as Ag [5], Mn [6] and B [7]. We have shown [5] that adding both Ag and B to the binary FePt alloy led to the direct formation of L1_0_ phase, with high coercivity and without the need for post-synthesis annealing of as-cast alloys. We have also previously shown that Mn addition in small amounts (36 at%) creates better stability of L1_0_ phase, and moreover, it promotes the occurrence of both binary FePt and ternary FeMnPt L1_0_ phases, making the alloys highly coercive [6,8]. Several other works reported show the intense focus on these alloys, as a pertinent alternative for the RE permanent magnets [9,10,11,12,13,14]. For instance, Sun et al. [9] investigated by X-ray absorption near-edge spectroscopy FeMnPt films and observed that by increasing Mn doping the ordering parameter of the L1_0_ phase decreases. The same effect was observed also by Xu et al. [10], who investigated epitaxial FeMnPt films and concluded that the increasing of Mn at 15 at% causes not only a decrease in the ordering parameter of the L1_0_ phase but also a decrease of the Curie temperature of the magnetic phase, accompanied by a decrease of the magnetic anisotropy of the films. Manoharan et al. [11] studied the magnetic and structural properties of highly ordered epitaxial Fe_50−x_Mn_x_Pt_50_ thin films and found a significant increase in the coercivity for a Mn content of 12 at%. They attributed the increase in coercivity to the tetragonal distortion, since they obtained a *c/a* ratio larger than the expected value for ferromagnetically-ordered Mn atoms in the Mn sublattice of the L1_0_ phase. As seen, there is a large preoccupation regarding the stability of the tetragonal L1_0_ phase in FeMnPt. Most of the studies regarding the correlation of magnetic performances to the stoichiometry of the FeMnPt alloys have been done theoretically by means of first principles DFT calculations [12,13] or ab initio [14]; therefore, there is a certain need to also assess experimentally the stability of the L1_0_ phase in FeMnPt alloys.

In the present paper we present a complex characterization and a structural stability study of the FeMnPt alloys system. For this, a mix of unconventional characterization methods, such as proton induced X-ray emission (PIXE) and high-temperature synchrotron radiation diffraction (HTSRD), and conventional methods, such as powder X-ray diffraction and SQUID magnetometry, were employed in order to derive the structure and magnetic properties and to prove the structural stability of the L1_0_ phase.

## 2. Experimental

For the method of synthesis of the FeMnPt alloys, ultra-rapid solidification from the melt was chosen to comply with the requirements of conserving the best chances for the formation of a tetragonal, ordered, L1_0_ phase in an alloy that typically crystallizes in a cubic, disordered A1. The ultra-rapid solidification from the melt, or melt spinning, is an out-of-equilibrium synthesis method which has the strong advantage that it preserves upon solidification; metastable phases that are only able to occur in the molten state, according to phase diagrams. As mentioned above, in order to improve chances of formation of L1_0_ phase via grain refinement at the nanoscale and to promote formation of a microstructure made of L1_0_ grains separated by less ordered intergranular regions, Mn addition in the binary FePt starting alloy has been considered. It has been also argued [10] that Mn addition in FePt binary alloy promotes the reduction of the temperature at which the disorder–order phase transition occurs. Moreover, in order to make these alloys more appealing from the raw material costs point of view, the Pt content has been reduced from around 50 at%, which is the classic content of the equiatomic FePt composition, to only about 35 at%. It is noteworthy that 35% is the lower limit for formation of L1_0_ phase, according to the FePt phase diagram [4]. We have adopted an experimental protocol in which a small amount of Pt is substituted by 8 at% Mn. A smaller amount would probably not result in the desired effect and a larger one may result in the creation of additional phases, which can be detrimental to the overall magnetic properties of the alloy.

Upon consideration of the above-mentioned arguments, ternary alloys with composition Fe_57_Mn_8_Pt_35_ were prepared by the high-speed melt solidification method, starting from basic materials with 99.9+% purity (Alfa Aesar GmbH, Karlsruhe, Germany). Elemental flakes have been melted in quartz crucibles in an induction furnace and the procedure was repeated three times to ensure a better homogeneity of the alloy. Ultra-rapid solidification from the melt was performed using a Buhler melt spinner facility in a protective Ar atmosphere. The melt was subsequently purged through a 5 mm nozzle onto a fast rotating (30 m/s), 50 cm diameter Cu wheel and solidified instantly (10^6^ K/s cooling rate) in the form of long, continuous, 5 mm wide, 30 microns thick, homogeneous ribbons. The as-cast ribbons were subsequently subjected to annealing in temperature-controlled oven, under vacuum (10^−4^ torr). For annealing, two sets of experimental parameters were chosen: 600 °C for 1 h and 700 °C for 1 h, with a heating rate of 5 K/min and cooling down to room temperature in vacuum.

The composition of the alloy has been verified using proton induced X-ray emission spectroscopy PIXE. PIXE analysis has been carried out using a 3 MeV proton beam generated with the aid of the 3 MV tandetron accelerator of the Horia Hulubei National Institute of Physics and Nuclear Engineering (NIPNE) Bucharest. The detection system includes a high purity Ge detector with an energy resolution of 150 eV at 5.9 keV for the X-rays. The steel target was mounted in the irradiation chamber at 45° with respect to the beam and the direction of both detectors. A thin surface barrier silicon detector was also placed in the chamber, at 135° with respect to the beam direction, in order to detect the backscattered protons, needed for spectra normalization. The X-ray, γ-ray and particle spectra were simultaneously collected and processed off-line.

In order to characterize the structural and magnetic properties, X-ray diffraction and SQUID magnetometry have been utilized. The structure of the as-cast and annealed ribbons was investigated by X-ray diffraction using a Bruker D8 Advanced diffractometer (Billerica, MA, USA). Diffraction patterns obtained were subsequently analyzed and fitted using a full-profile Rietveld-type analysis, powered by MAUD software [15]. The structural stability and phase evolution with temperature have been monitored using synchrotron X-ray diffraction. To determine the evolution with temperature of the phase structure in our alloy and to follow the disorder–order phase transformation, a temperature-dependent synchrotron X-ray diffraction experiment, conducted between 50 °C and 800 °C, was performed at the Materials Characterization by X-ray diffraction (MCX) beamline, Elettra Synchrotron Facility, Trieste, Italy, using monochromatic radiation (λ = 0.0826 nm).

Magnetometry measurements have been performed in order to determine the magnetic properties of the as-cast and annealed alloys. Initial magnetization vs. applied field and the hysteresis loops were measured at 5 K and 300 K with a magnetic field applied parallel to the ribbons plane, using a Quantum Design SQUID magnetometer (Quantum Design GmbH, Darmstadt, Germany) in RSO (reciprocate sample option) mode.

## 3. Results and Discussions

The chemical composition of the melt-spun ribbons, prepared as mentioned before, has been verified using proton induced X-ray emission spectroscopy, PIXE. Proton-induced, or more generally particle-induced X-ray emission is an elemental analysis technique which uses a highly energetic beam of heavy charged particles (usually protons of kinetic energy between 1 and 4 MeV) to produce element-specific X-ray emission from solid samples. Compared with more classical compositional analysis approaches such as energy-dispersive X-ray spectroscopy, PIXE has the advantage of detection limits of 0.1–10 mg/kg (or 10^−5^–10^−7^) concentration level for low Z elements, depending on the sample material and its thickness. 

**Figure 1:**
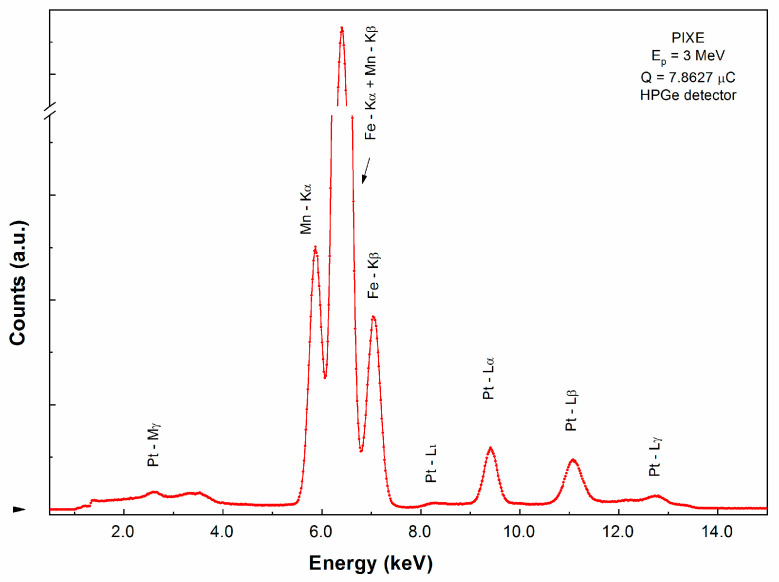
Proton-induced X-ray emission spectrum of as-cast Fe_57_Mn_8_Pt_35_ alloy.

PIXE experiments have been performed at the 3 MV Tandetron of NIPNE using a 3 MeV proton beam. Proton beam current incident on the target was between 1 and 5 nA and the exposure time was about 30 min. The targeted sample was placed in the center of the irradiation chamber, perpendicular on the beam direction. The size of the proton beam spot on the target was about 1 mm^2^. The X-ray detection was performed with a high-purity Ge detector from Ortec, with 6 mm active diameter and 6 mm active depth, having a 12.7 mm Be window and performing with an energy resolution of 150 eV at 5.9 keV. The detector was mounted inside the reaction chamber and oriented at 45° with respect to the beam direction. The beam transport tubes and target chamber were maintained in a high vacuum (10^−5^ mbar) during irradiation. The Guelph PIXE program has been used for the quantitative analysis [16]. The PIXE spectrum of the as-cast sample is shown in Figure 1. Based on the relative intensity of the emission lines K, L and M, the average experimental composition was calculated to be Fe 58.05%, Mn 7.06% and Pt 34.89% as seen in Table 1. These values are very close to the nominal composition for the alloy: Fe_57_Mn_8_Pt_35_. 

**Figure 2:**
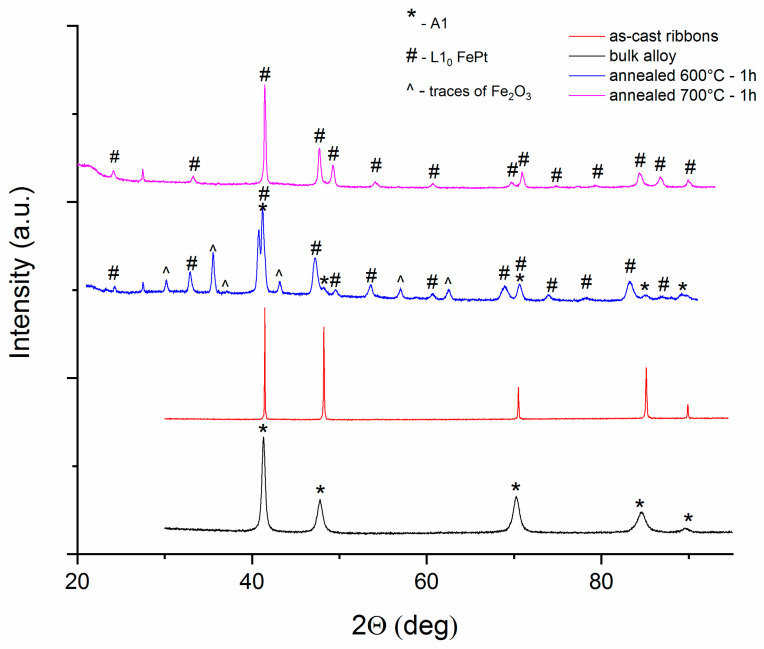
X-ray diffractogram of Fe_57_Mn_8_Pt_35_ in bulk state, as cast ribbons and ribbons annealed at 600 °C-1 h and 700 °C-1 h.

The structural characterization of the sample has been performed using X-ray diffraction. The X-ray diffractograms for the bulk alloy, i.e., the alloy before melt spinning, the as-cast and annealed ribbons are presented in Figure 2. In the case of the bulk sample, obtained directly from the melt, the X-ray diffractogram shows Bragg lines that are attributed to the A1, (Fe,Mn)Pt, body-centered-cubic phase. These lines are quite wide, typical for intermetallic solid-solution diffractograms. The as-cast ribbons present Bragg lines at almost same position as in the bulk alloy, but much narrower. Here, it appears that the cubic phase is better formed, with larger crystallographically coherent domains, belonging to the body-centered-cubic symmetry of A1 phase. The two diffractograms, belonging to the two annealed samples, have different allures. While the bulk and as-cast samples have been investigated between 30° and 95° in 2θ, for the annealed samples this interval has been enlarged, from 20° to 95° in 2θ, in order to spot also potential L1_0_-belonging superlattice peaks. As it turns out, that was beneficial, as several other Bragg lines have indeed been observed for both diffractograms, for the samples annealed at 600 °C and 700 °C. Full-profile Rietveld-type analysis performed on the diffractograms has revealed that the additional peaks could be unambiguously identified as belonging to the tetragonal L1_0_ FeMnPt phase, for the two annealed samples. Bragg lines with smaller intensity found only in the sample annealed at 600 °C have been attributed to an Fe oxide phase. It is worthwhile mentioning that the oxide has not been seen in the sample annealed at 700 °C, so it could have been due to slight oxidation during one annealing procedure. The nature of the L1_0_ phase in the ternary alloy is slightly different from the one occurring in the binary compound. If Mn is added to FePt, then the tetragonal L1_0_ structure unit cell consists of alternate stacking of layers of Pt atoms alternating with layers originally containing only Fe atoms. Added Mn atoms are, however, distributed randomly on crystallographic positions that were initially occupied by Fe atoms. It is inferred that the presence of Mn atoms in the structure facilitates the occurrence of tetragonal L1_0_ phase by creating vacancies among the Fe atoms positions which allow rearrangement of the fastest atoms during the structural phase transformation, and reinforcement of grain boundaries and intergranular areas by segregation of excess Mn atoms which do not accommodate the L1_0_-available crystallographic positions. Due to slight lattice distortion, the unit cell of ternary L1_0_ phase would be different from its binary counterpart. In fact, co-existence of the two L1_0_ phases, binary and ternary, has been observed through atomic resolution TEM and electron diffraction patterns [6] in other FeMnPt alloys.

It is known that the most intense Bragg lines, corresponding to the reflections (111) and (200) of the cubic A1 and tetragonal L1_0_ phases, are relatively close in terms of angular position. Therefore, sometimes due to both the spectral instrumental broadening of the X-ray beam and the broadening of the Bragg reflections due to grain sizes, the difference in angular position width of the spectral Bragg line is too small for accurate deconvolution of the two phases. However, the L1_0_ phases present several additional Bragg lines, the so-called superlattice peaks, which constitute the specific signature of the presence of these phases in X-ray diffractograms. Two examples of such superlattice peaks are the reflections (001) and (110), located approximately at 24° and 32°, respectively, in 2θ (for the wavelength we used, of Cu Kα - 1.54Å).

The disorder–order phase transformation with formation of L1_0_ phase is therefore evidenced through the XRD results, mainly by two distinct features: (a) both annealed samples diffractograms show splitting of the main Bragg lines (111) and (200), located around 41.5° and 48.3°, showing clear separation of the Bragg lines of tetragonal L1_0_ and cubic phase respectively, and (b) the occurrence of several superlattice Bragg lines unambiguously attributed to the tetragonal L1_0_ phase. This indicates the formation of the tetragonal phase which co-exists with the initial cubic phase, at 600 °C annealing temperature.

The ribbons annealed at 700 °C–1 h show a XRD diffractogram wherein only one structural phase is observed: the tetragonal L1_0_ phase. Contrary to the case of 600 °C annealing, the Bragg lines of Fe oxide were not observed anymore, and moreover, neither were the Bragg lines of the cubic A1 phase. This indicates that for 700 °C annealing, the disorder–order phase transformation is finished, the tetragonal phase formation is completed and the cubic phase is no longer detectable in the annealed sample.

The lattice parameters and mean grain size, assimilated here with the volume-averaged crystallographically coherent domains, as obtained after full profile Rietveld-type fitting of the XRD experimental diffractograms, are given in Table 2. Using the half width of the diffraction lines (FWHM) average grain size was estimated by Scherrer’s method. It can be seen from the analysis of Table 2 that the grain size of the bulk sample obtained from the melt is about 32 nm, which indicates that the cubic phase has a nanocrystalline structure. Upon rapid solidification followed by melt spinning of the alloy, a recrystallization of the bulk nanocrystalline structure occurs; therefore, the as-cast ribbons have a larger average grain size of about 130 nm. After subsequent annealing, the disorder–order phase transformation is promoted and results in the formation of a L1_0_ tetragonal phase. This is accompanied by a structural refinement through atom mobility which causes a fracturing of the large crystallites of cubic solid solution observed initially in the bulk alloy, into grains of several nm diameter. Consequently, through Mn segregation during L1_0_ grains nucleation, intergranular Mn-rich patches are formed. For the sample annealed at 600 °C for 1 h, the mean grain sizes are around 32 nm for A1 and 28 nm for L1_0_ phases, respectively. For annealing at 700 °C, the structure evolves from a nucleation to growth mechanism, and single phase L1_0_ is obtained, having an average grain size of about 45 nm (microstructure coarsening).

In order to monitor the evolution with temperature of the phase structure in the Fe_57_Mn_8_Pt_35_ sample and to follow the disorder–order phase transformation, a temperature-dependent synchrotron X-ray diffraction experiment (between 50 and 800 °C) was performed in controlled atmosphere in the dedicated furnace with the MCX beamline. The furnace is composed of a main cylindrical vacuum room divided by a water-cooled Cu diaphragm that acts as a thermal barrier between a hot and a cold zone. The hot area contains the heating elements, while the cold zone contains the sample alignment and the gas flow system. The detector consists of a curved magnetic support that can move and can support a photographic plate that allows one to acquire multiple XRD patterns in the same image. Photographic plate is fixed so that it can be exposed to a film of 20 cm in a 2θ range of about 130°. The temperature measurement is accomplished using a thermocouple. The diaphragm has a 5 mm aperture in the center to allow the capillary to enter in the heating elements. The capillary base is mounted on a support which is located in the cold zone. The entire vacuum chamber is perpendicular to the beam, which allows easy access to the cold zone for replacement and alignment of the capillary. During measurements in the chamber the vacuum is 10^−3^ bar to avoid air scattering and heat loss. The diffracted beam exits the chamber through an interchangeable slit, aligned with the ceramic gap of the heating element.

The diffraction experiment was performed starting with heating from 25 °C and recording the first diffractogram at 50 °C with a heating rate of 10 K/min. X-ray diffractograms have been recorded from 50 to 800 °C with a 50 °C step. The heating during measurements were carefully performed; upon reaching the set temperature value, additional 2 s settle time was allowed for temperature stabilization at the desired value to within 1 °C. Total exposure time of the sample at a given temperature was about 1 min.

**Figure 3:**
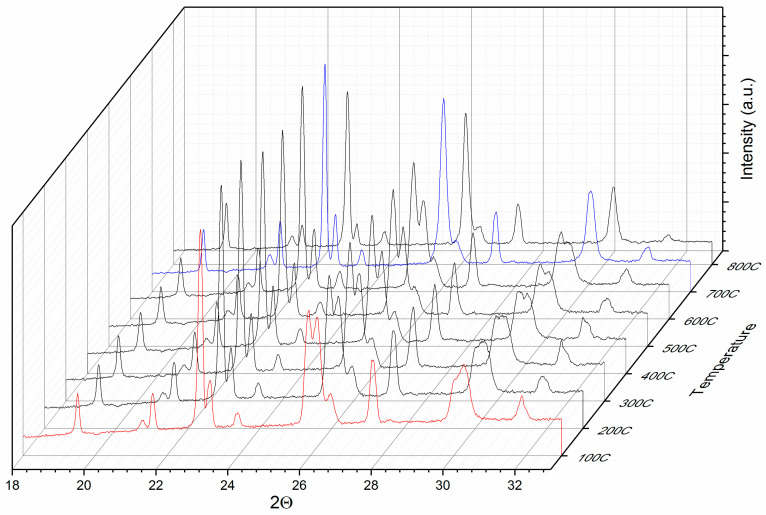
3D representation of selected synchrotron XRD data taken between 100 and 800 °C for the as-cast sample.

Figure 3 presents a 3D collection of selected diffractograms recorded at temperatures between 100 and 800 °C. Since not all of the recorded diffractograms could be represented for reasons of clarity of the representation, we chose to depict only those between 100 and 800 °C with a 100 °C step.

It can be observed that the Bragg lines observed are consistent throughout the whole range of investigated temperatures and similar features are observed for all investigated temperatures. The observed Bragg lines are similar for all the investigated temperatures with a certain position shifting, due to the lattice parameters increasing with temperature. Particular aspects are, however, to be noted, especially for the reflections situated at around 26° and 30°. During the disorder–order phase transformation, some of the Bragg lines of the cubic phase split due to tetragonal distortion. Upon occurrence of L1_0_ phase, these Bragg lines split; for example, (200) of the cubic phase into (200) and (002) of the tetragonal phase.

**Figure 4:**
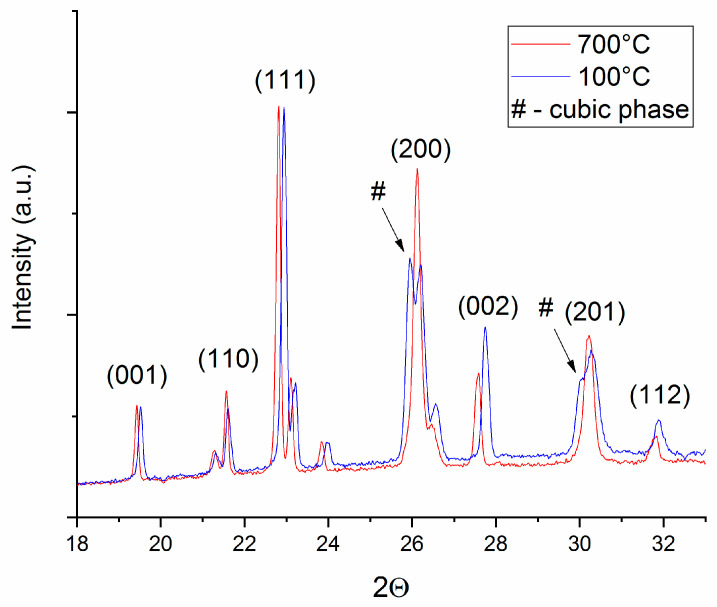
Details of the synchrotron XRD data recorded at 100 °C (blue) and 700 °C (red).

The more obvious the splitting, the more significant the phase transformation and the more abundant the formed L1_0_ phase. It is worthwhile noticing that in the diffractograms recorded at 50 and 100 °C, there is an obvious splitting of the peaks at 26° and 30° in 2Θ. This splitting disappears at 700 °C. For clarity we have plotted in Figure 4 only the 100 and 700 °C diffractograms, zoomed in on the area of interest. It can be seen here more clearly that the split part of the blue Bragg lines, at 100 °C, indicated with an arrow on the graph, is completely missing at 700 °C. This represents evidence that while the disorder–order phase transformation is gradual over the investigated temperature range, at 700 °C this transformation is completed and no more cubic phase is observed in the 700 °C diagram (red) since the splitting is no more observed in the graph for these particular Bragg lines.

**Figure 5:**
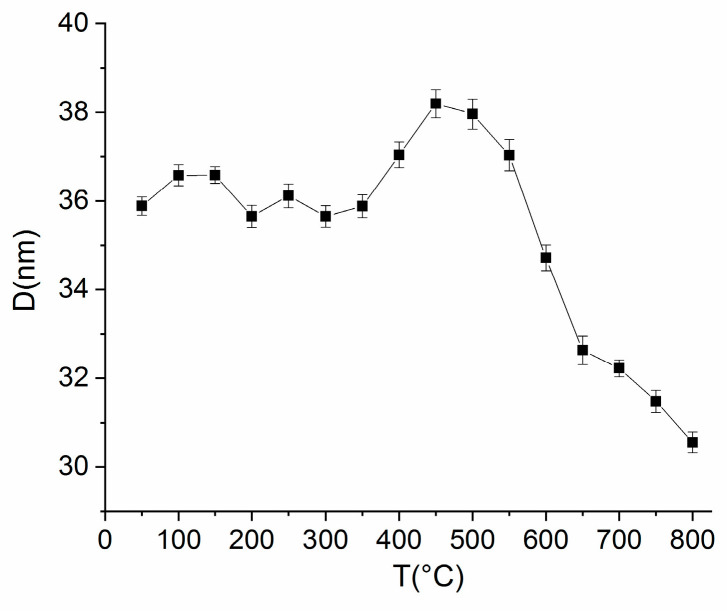
Average grain size of the L1_0_ phase as a function of temperature of XRD measurement.

**Figure 6:**
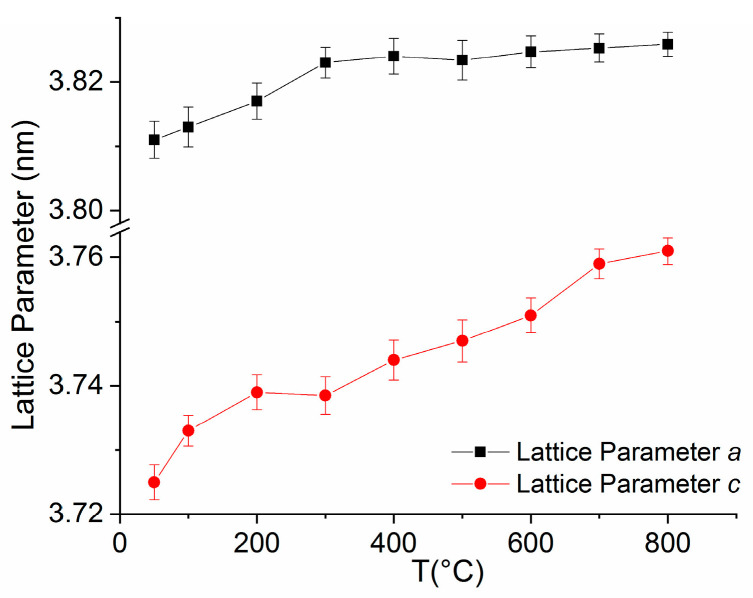
Lattice parameters of the tetragonal L1_0_ phase, *a* and *c*, vs. temperature of XRD study.

The XR diffractograms have been fitted using full profile Rietveld-type analysis. The attribution of Bragg lines is facilitated by the good spectral resolution of the synchrotron X-rays, and therefore, peaks of the same reflections (for instance (200) or (220)) belonging to different phases, cubic and tetragonal, which are otherwise convoluted, are clearly observed experimentally and easier to identify correctly. Lattice parameters have been calculated for the L1_0_ phase for selected diffractograms in the 50–800 °C range. From the full width at half maximum of the main Bragg line (111) of L1_0_ phase, resulting from the fitting, we have calculated the average grain size assimilated with the crystallographically coherent domain. These structural parameters give information about the structural stability of the L1_0_ phase over a large temperature interval, as the stability of the hard magnetic phase is essential for its use in many applications, especially for magnets operating in extreme conditions, such as jet engines, turbines or in automotive engines. Figure 5 depicts the average grain size of the L1_0_ phase as a function of temperature while Figure 6 presents the lattice parameters of the tetragonal L1_0_ phase, *a* and *c*, vs. temperature.

The average grain size dependence vs. temperature has a quite constant behavior between 50 and 400 °C, with some small oscillations around 36 ± 0.3 nm value, fluctuations that are within the estimation errors. A small increase was observed with a maximum of 38 ± 0.3 nm at about 450 °C, and then, starting at 550 °C the grain size diminished until the end of the investigated temperature range down to 31 ± 0.3 nm. This decrease coincides with the temperature range where the disorder–order phase transformation comes to an end and there is no more co-existence with the cubic phase. The most striking result is, however, the absence of grain-growth that would have been expected with a temperature increase. It seems that the L1_0_ structure at the nanoscale is preserved down to average grain size fluctuations of only a few nanometers throughout the whole temperature interval investigated. This brings further proof that one of the roles of Mn addition is to inhibit the grain growth in the sample, this being preserved up to the highest temperature investigated (800 °C).

Structural parameters are calculated using fitting results from MAUD. The full-profile fitting yielded good correspondence to the experimental data, as proven by the refined weight indexes and GoF (σ values) resulting from the fit. Typical values for MAUD fitting of the X-ray diffractograms are: σ = 1.4; R_w_ (%) = 2.28; R_wnb_ (%, no bkg) = 7.52; R_b_ (%) = 1.42; R_exp_ (%) = 1.15. The lattice parameters vs. temperature variation (Figure 6) show for the *a* parameter a very slight and continuous increase with temperature of about 3.6% throughout the whole temperature range. The *c* parameter has the same tendency with a slightly larger increase of about 9.6% throughout the whole temperature range, being a consequence of slight imbalance towards more out-of-plane unit cell expansion with temperature, a phenomenon that has been observed also in FeCoPt alloys [17]. As a consequence, the ordering parameter *c/a* ratio varies between 0.977 and 0.983 for the 50–800 °C temperature range. This remarkably high degree of ordering exceeds the values reported in well-ordered L1_0_ FeMnPt films deposited at 550 °C [13] or in films annealed at 600 °C for 1 h [18]. The absence of grain growth combined with the thermal stability of the L1_0_ structure and the very high degree of ordering in these alloys further confirms that the addition of Mn to FePt is efficient at suppressing diffusion processes and completely inhibits the grain-growth of the L1_0_ FeMnPt phase up to 800 °C, which is quite encouraging in view of potential applications of magnetic materials in extreme-condition technologies.

**Figure 7:**
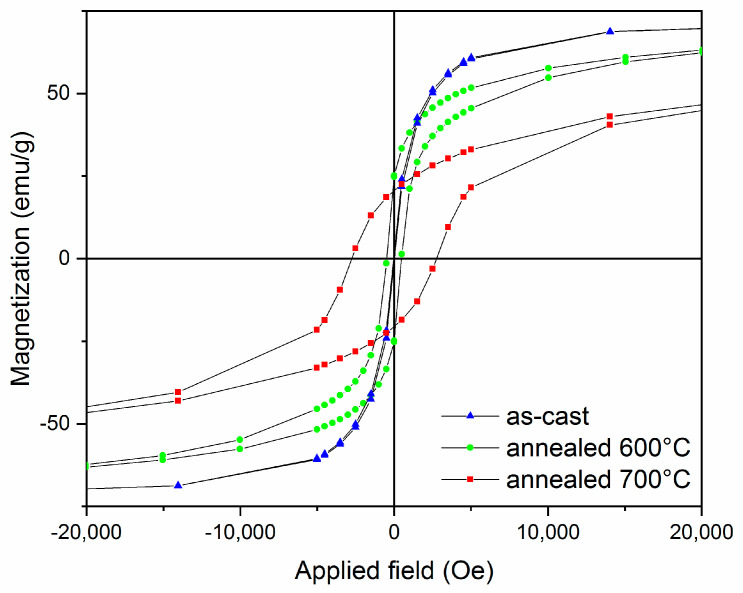
Hysteresis loops for the as-cast and annealed Fe_57_Mn_8_Pt_35_ sample.

Magnetic properties of samples submitted to static annealing at 600 and 700 °C have been measured with SQUID magnetometry. Hysteresis curves have been measured at 300 K in applied magnetic fields up to 5 Tesla, applied perpendicularly to the ribbons plane (Figure 7). The hysteresis loop for the as-cast sample showed typical soft ferromagnetic behavior with ab approach to saturation at low applied fields and high saturation magnetization (70 emu/g) with virtually no coercivity (only 95 Oe coercive field). The sample annealed at 600 °C showed similar soft magnetic features with slightly smaller saturation magnetization (around 67 emu/g) but increased coercivity (470 Oe). The increase of the coercive field is related to the presence of the hard magnetic L1_0_ phase co-existing with the soft magnetic, cubic A1, which is the only phase present in the as-cast sample. The magnetic data are thus in agreement and confirm the structural data obtained on the as-cast and annealed sample. Co-existence of cubic and tetragonal phases in the alloy lead to modifications in the hysteresis loop of the annealed samples. Cubic phase is soft magnetic with high magnetization, while the tetragonal phase is hard magnetic with high coercivity. An optimal coupling of these two phase would mean observing a hysteresis loop exhibiting both features: high magnetization and high coercivity, ultimately leading to a maximum energy product (BH)_max_ superior to any of the constituent phases. If the openness and overall shape of the hysteresis loop is typical of a single magnetic phase, i.e., no inflection points being observed in the demagnetization region of the loop (second quadrant), it means that the two phases are fully exchange coupled, as is the case for exchange spring magnets. This is what was obtained also in our annealed alloys, as the lack of inflection points show that the two observed phases, hard and soft magnetic, are fully exchange coupled in the intermediate annealing regime (600 °C). This may potentially lead to the creation of a Fe-Mn-Pt exchange-coupled nanocomposite magnet where both soft and hard magnetic phases coexist and may yield good magnetic performances, comparable with existing permanent magnets. A strong increase of the coercivity was observed for the sample annealed at 700 °C; the coercive field reached 2.8 kOe and a saturation magnetization of about 49 emu/g. This is, again, consistent with the structural data, for which we noticed that when the disorder–order phase transformation was complete, the cubic A1 phase was no longer observed and the hard magnetic L1_0_ tetragonal phase was the only magnetic phase present in the sample. The findings confirm, thus, the features observed through structural data and point out the beneficial effect of Mn addition in view of stabilizing the L1_0_ phase structure on a large temperature range, needed for technological applications in extreme operation conditions.

## 4. Conclusions

In order to assess the effect of Mn addition on the structural stability of L1_0_ FePt phase, a Fe_57_Mn_8_Pt_35_ granular alloy has been synthesized in the shape of melt-spun ribbons and subsequently annealed at 600 °C and 700 °C for promoting the formation of single phase L1_0_ tetragonal, hard magnetic phase. A mix of unconventional and classic characterization techniques has been employed for compositional, structural and magnetic analysis of the as-cast and annealed samples. Proton-induced X-ray emission spectroscopy was employed in order to assess the compositional effects in the ternary alloy while powder X-ray diffraction followed by full profile Rietveld-type analysis was used for structural and crystallographic analysis. The disorder–order phase transformation and the stability of the hard magnetic L1_0_ phase were monitored over a large temperature range (50–800 °C) using temperature-dependent synchrotron X-ray diffraction. It was found that there is a large interval of stability of the L1_0_ phase formed upon the disorder–order phase transformation. The Mn addition was found to promote a higher ordering of the L1_0_ phase, as proven by the remarkably high tetragonality ratio *c/a* parameter. The absence of crystal growth and unit cell expansion up to the highest temperature investigated reveals that the Mn addition stabilizes the formed L1_0_ structure further. Magnetic characterization has revealed, in total agreement with the structural data, that a strong coercive field is obtained for a sample wherein single phase hard magnetic tetragonal L1_0_ exists. These findings are very promising in view of use of such materials as a future class of rare earth free permanent magnets that are capable of operating in stable and reproducible manner in applications that require extreme operation conditions.

## Figures and Tables

**Table 1:** PIXE results obtained on as-cast FeMnPt sample.

Element.	Atomic Mass M	Concentration PIXE (%)	Standard Deviation σ (%)	Concentration Ratio (PIXE)	Atom No. Ratio (PIXE)	Chemical Formula (PIXE)
**Mn**	54.938	**3.72**	0.83	0.057	**0.202**	**7.06**
**Fe**	55.847	**31.07**	0.52	0.476	**1.664**	**58.05**
**Pt**	195.08	**65.24**	0.33	1.000	**1.000**	**34.89**

**Table 2:** X-ray diagrams fitting results for the sample Fe_57_Mn_8_Pt_35_.

Samples.	Lattice Parameters (Å)	Size (nm)
Fe_57_Mn_8_Pt_35_ - bulk	Cubic A1 a = 3.790 ± 0.006	D = 32 ± 1.3
Fe_57_Mn_8_Pt_35_ - ribbons	Cubic A1 a = 3.774 ± 0.003	D = 130 ± 2.6
Fe_57_Mn_8_Pt_35_-600 °C-1 h	Tetragonal L1_0_	D = 28 ± 0.6
	a = 3.860 ± 0.012	
	c = 3.697 ± 0.009	
	Cubic phase A1	D = 33 ± 0.5
	a = 3.837 ± 0.009	
	Cubic phase Fe_2_O_3_	D = 52 ± 3
	a = 8.378 ± 0.011	
Fe_57_Mn_8_Pt_35_ 700 °C-1 h	Tetragonal L1_0_ a = 3.809 ± 0.007 c = 3.699 ± 0.009	D = 45 ± 1.4
